# Development and validation of prognostic and diagnostic models utilizing immune checkpoint-related genes in public datasets for clear cell renal cell carcinoma

**DOI:** 10.3389/fgene.2025.1521663

**Published:** 2025-03-04

**Authors:** Bin Zhao, Shi Fu, Yuanlong Shi, Jinye Yang, Chengwei Bi, Libo Yang, Yong Yang, Xin Li, Zhiyu Shi, Yuanpeng Duan, Zongyan Luo, Guoying Zhang, Jiansong Wang

**Affiliations:** ^1^ Department of Urology, Peking University Cancer Hospital Yunnan, Yunnan Cancer Hospital, The Third Affiliated Hospital of Kunming Medical University, Kunming, Yunnan, China; ^2^ Department of Urology, The Second Affiliated Hospital of Kunming Medical University, Kunming, Yunnan, China

**Keywords:** clear cell renal cell carcinoma (ccRCC), immune checkpoint-related genes, EGFR, TRIB3, ZAP70, CD4, prognosis, diagnosis

## Abstract

**Background:**

Clear cell renal cell carcinoma (ccRCC) is the most prevalent subtype of renal cell carcinoma, and immune checkpoint regulator-based immunotherapy has emerged as an effective treatment for advanced stages of the disease. However, the expression patterns, prognostic significance, and diagnostic value of immune checkpoint-related genes (ICRGs) in ccRCC remain underexplored. This study utilized large-scale ccRCC datasets from The Cancer Genome Atlas (TCGA), Gene Expression Omnibus (GEO), and the International Cancer Genome Consortium (ICGC) to analyze ICRGs and develop a prognostic and diagnostic model, which was validated using quantitative PCR in clinical samples from ccRCC patients.

**Methods:**

RNA-seq data and clinical information were retrieved from TCGA, ICGC, and GEO databases. Differentially expressed genes (DEGs) were identified, and immune checkpoint-related genes (DICRGs) were selected by intersecting DEGs with ICRGs, followed by validation in independent datasets. Univariate and multivariate Cox regression analyses were used to develop the prognostic model. Protein expression of key genes was validated through immunohistochemistry (IHC) using data from the Human Protein Atlas (HPA). qRT-PCR confirmed gene expression levels in ccRCC and normal kidney tissues. Diagnostic models were constructed using machine learning, and functional enrichment and immune infiltration analyses were performed.

**Results:**

Fourteen DICRGs were identified, with four (*EGFR*, *TRIB3*, *ZAP70*, and *CD4*) showing prognostic significance in Cox analyses. IHC revealed high expression of these genes in ccRCC tissues, and qRT-PCR confirmed increased expression of *EGFR*, *TRIB3*, and *CD4*, while *ZAP70* expression showed no significant change. A prognostic risk score was developed based on gene expression levels. Functional analysis identified enriched pathways related to organic anion transport and metabolism, while immune infiltration analysis revealed associations between *ZAP70*, *CD4*, and risk scores.

**Conclusion:**

This study establishes a prognostic model for ccRCC based on four ICRGs, providing valuable insights into the molecular mechanisms underlying prognosis and diagnosis in ccRCC.

## 1 Introduction

Renal cell carcinoma (RCC) has emerged as one of the most prevalent genitourinary tumors, ranking second only to prostate and bladder cancers in incidence, and stands as the deadliest malignancy affecting the urinary system (Xia et al., 2022). Clear cell renal cell carcinoma (ccRCC) represents the predominant pathologic subtype, comprising 70%–85% of renal cancer cases, with a notably high occurrence (Chen et al., 2019). Owing to the subtle clinical manifestations of early-stage renal cancer, metastasis is detected in approximately 25% of patients upon diagnosis, and over 20% of patients experience distant metastasis following radical surgery for renal cancer (Akhtar et al., 2019; Linehan and Ricketts, 2019), leading to an unfavorable prognosis. Furthermore, ccRCC exhibits insensitivity to conventional radiotherapy and chemotherapy, is predisposed to drug resistance, and lacks dependable prognostic biomarkers, resulting in disease progression within a two-year timeframe for most tumors (Motzer et al., 2015). Despite extensive research into cancer development mechanisms, the etiology and carcinogenesis of RCC remain elusive. Hence, further investigation into novel and efficacious prognostic biomarkers is imperative to enhance the prognosis of ccRCC patients.

Immunotherapy has garnered attention in renal cancer since 2015, when the use of the immune checkpoint inhibitor Nivolumab was confirmed for advanced renal cancer (Motzer et al., 2022; Lee et al., 2022). In recent years, immunotherapy has made significant strides in treating various tumors (Liu et al., 2022). It has been demonstrated that immune checkpoint inhibitor therapy promotes active host immune responses through diverse mechanisms, including gene mutation, epithelial-mesenchymal transition, and metabolism (Marei et al., 2023). Presently, several guidelines have endorsed targeted combination immunotherapy or dual immunotherapy for advanced kidney cancer (Gebrael et al., 2023; Rini et al., 2019; Rustum et al., 2023). According to the International Metastatic Renal Cell Carcinoma Database Consortium (IMDC) risk stratification, all patients with metastatic ccRCC requiring first-line systemic therapy should receive immune checkpoint inhibitor (ICI) therapy in combination with a vascular endothelial growth factor receptor tyrosine kinase inhibitor (VEGFR) or two immune checkpoint inhibitors (ICIs) for patients with intermediate-risk or high-risk disease (Ahmadie et al., 2022). Currently, immune checkpoint inhibitor therapy primarily targets the immune escape mechanism of tumor cells, but few studies have systematically analyzed the expression pattern of immune checkpoint-related genes (ICRGs) in ccRCC.

In this study, we developed a robust prognostic model for ccRCC patients using transcriptional data from TCGA and other public databases, focusing on immune checkpoint-related genes (ICRGs). The model’s validity and reliability were confirmed across numerous independent external datasets. Additionally, we conducted qRT-PCR experiments to validate the expression levels of four identified DICRGs in each group, highlighting their potential as prognostic biomarkers and therapeutic targets. This study not only offers a foundation and new reference for ccRCC treatment but also provides insights into the molecular mechanisms underlying ccRCC prognosis. The prognosis-related DICRGs identified herein lay a theoretical groundwork for enhancing diagnosis and treatment strategies for ccRCC patients.

## 2 Materials and methods

### 2.1 Sources of information

RNA sequences and clinical data from 530 ccRCC patients and 72 normal kidney tissues were acquired from the Tumor Genome Atlas (https://portal.gdc.cancer.gov). Additionally, ninety-one RNA-SEQ datasets containing survival data for ccRCC patients were obtained from the International Cancer Genome Consortium (ICGC) database (https://dcc.icgc.org) for external validation. The GSE53757 and GSE15641 datasets were retrieved from the Gene Expression Overview database (https://www.ncbi.nlm.nih.gov/geo). The GSE53757 dataset (GPL570 [HG-U133_Plus_2] Affymetrix Human Genome U133 Plus 2.0 Array) comprised 72 normal samples and 72 ccRCC samples, while the GSE15641 dataset (GPL96 [HG-U133A] Affymetrix Human Genome U133A array) included 23 normal and 32 ccRCC samples. Differential gene analysis was conducted using the GSE53757 dataset, while the performance of the diagnostic model and the expression of risk model genes were validated using the GSE15641 dataset. Subsequently, 282 immune checkpoint-related genes (ICRGs) were identified from the literature (Zhao et al., 2021).

### 2.2 Screening of differential ICRGs (DICRGs)

Differentially expressed genes (DEGs) between ccRCC and normal groups in TCGA, as well as ccRCC and normal groups in GSE53757, were identified based on P - value < 0.05 and |log_2_FC|>1 criteria utilizing the R language package “limma” (version 3.42.2) (Ritchie et al., 2015). Heatmaps were generated using the pheatmap package (version 1.0.12), while box plots were created using the ggplot2 package (version 3.3.2) (Ito and Murphy, 2013) In this study, the method section employs the Wilcoxon rank - sum test (P - value <0.05) to compare differences among different groups and evaluate the significance of differences in gene expression levels or other variables when creating boxplots.

### 2.3 Construction and evaluation of predictive models

The expression data of DICRGs were obtained from the ccRCC sample expression data in TCGA. This data was then integrated with clinical information to derive the clinical expression profiles of 526 ccRCC samples, excluding those with missing survival data. Subsequently, in order to evaluate the generalization ability of the model, the 526 samples were divided into a training cohort and a test cohort at a ratio of 6:4 (training cohort = 316, test cohort = 210). In the training cohort, a univariate Cox algorithm was employed to identify prognostically relevant DICRGs with significance level p < 0.2 (Ye et al., 2021). Following this, a multivariate Cox analysis was conducted using the step function. The Cox model formula utilized was:[ h(t/X) = h_0(t) exp (β_1 X_1 + β_2 X_2 + …… + β_p X_p) ] where (h_0(t)) represents the baseline risk function at time (t) when all variables are zero, (X_1, X_2, … , X_p) denote the influencing factor variables, and (β_1, β_2, … , β_p) refer to the regression coefficients. Patients were then scored based on the risk coefficients and expression values derived from the multivariate Cox analysis. The surv_cutpoint function within the survival package was utilized to determine the optimal cutoff value for the continuous independent variable of the survival profile (train:0.85; test:1.42; ICGC:1.18). Based on this cutoff, patients were categorized into high-risk and low-risk groups. Survival analyses were conducted using the Survival package (version 3.2-7) (N and Lee, 2019). Additionally, the SurvivalROC package (version 1.0.3) (Robin et al., 2011) was employed to calculate the area under the curve (AUC) values of the ROC curves, serving as a measure of the predictive model’s accuracy. Finally, the prognostic model underwent validation using both a test cohort and an external validation cohort.

### 2.4 Bioinformatic validation of expression levels of prognosis-related DICRGs

In order to verify the expression level of prognosis-related DICRGs. First, the expression levels of the four prognosis-related DICRGs were validated using the Wilcoxon test method in the GSE15641 dataset. Then, the protein expression levels of DICRGs in both ccRCC and adjacent normal tissues were confirmed through immunohistochemical staining. Immunohistochemistry (IHC) results were acquired from the Human Protein Atlas database (HPA) available at https://www.proteinatlas.org/.

### 2.5 Real-time quantitative polymerase chain reaction (qRT-PCR)

#### 2.5.1 Sample collection

Ten pairs of cancerous and paracancerous tissues were obtained from ccRCC patients undergoing nephrectomy at Yunnan Cancer Hospital in Kunming, China. All patients were diagnosed with clear cell renal cell carcinoma based on postoperative pathology. The study protocol was approved by the Ethics Committee of Yunnan Cancer Hospital (Approval No. SLKYLX2022258).

#### 2.5.2 Total RNA extraction

Ten pairs of tissue samples were divided into two groups: 10 samples constituted the normal group, while the remaining 10 samples formed the experimental group. For each sample, 50 mg of tissue was taken, and 1 mL of TRIzol reagent was added. After complete homogenization, the mixture was left on ice for 10 min to ensure cell lysis. Subsequently, 300 uL of chloroform was added, vigorously shaken for 30 s, and left at room temperature for 10 min to allow for phase separation. The mixture was then centrifuged at 12,000 g for 15 min at 4°C, resulting in the separation of liquid into three layers, with the RNA retained in the upper colorless aqueous phase. The upper aqueous phase was carefully transferred to another EP tube, and an equal volume of ice-cold isopropanol was added. After inversion and mixing, the mixture was allowed to stand for 10 min and then centrifuged at 12,000 g, 4°C for 10 min, yielding a white RNA precipitate at the bottom of the tube. The supernatant was discarded by gently tilting the tube, and the mouth of the tube was dried using absorbent paper. To the precipitate, 1 mL of 75% ethanol was added, followed by gentle inversion to facilitate precipitation floating. After a 2-min rest, centrifugation at 7,500 *g*, 4°C for 5 min was performed to further settle the precipitate, repeating this step twice. The supernatant was then discarded, and the centrifuge tube was inverted on absorbent paper. Careful aspiration of the remaining liquid with a 10ul tip was carried out, followed by natural drying for 20 min or blow drying in an ultra-clean bench to remove ethanol and water residue, rendering the RNA precipitate transparent. Subsequently, add 20–50 μL of RNase-free water to the dried RNA precipitate and let it stand for 15 min to ensure complete dissolution of the RNA. Take 1 μL for concentration detection with NanoDrop, recording the RNA purity/concentration to determine the sample amount for the subsequent reverse transcription step. Subsequently, the remaining RNA should be either reverse transcribed immediately or frozen and stored in the refrigerator at −80°C.

#### 2.5.3 RNA detection

Utilize 1 μL of RNA for detection using NanoPhotometer N50, with the results detailed in Supplementary Table S1 *.*

#### 2.5.4 mRNA reverse transcription

The **SureScript First-**strand cDNA Synthesis Kit from Xavier was employed as follows: extract the components of the reverse transcription **kit, al**low them to melt at room temperature, briefly centrifuge, place on ice, and then 4 μL of Reaction Buffer and 1 μL of Primer were added. After centrifugation, an additional 4 μL of Reaction Buffer, 1 μL of Primer, 1 μL of SweScript RT I Enzyme Mix, 5 μg of RNA, and 9 μL of Nuclease-Free Water were added while on ice The process of reverse transcription was then carried out on a standard PCR instrument.

#### 2.5.5 On-line assay

The aforementioned reverse transcription product, cDNA, was diluted 10-fold with RNase/DNase-free ddH2O. Subsequently, 3 μL of cDNA, 5 μL of Universal Blue SYBR Green qPCR Master Mix, 1 μL of Forward primer, and 1 μL of Reverse primer were added for the qPCR reaction. Following a brief centrifugation, 40 cycles of the reaction were executed in a CFX96 Real-Time Quantitative Fluorescent PCR Instrument to generate amplification and melt curves, and to determine the Ct values. The amplification process included an initial pre-denaturation step at 95°C for 1 min, followed by 40 cycles of denaturation at 95°C for 20 s, annealing at 55°C for 20 s, and extension at 72°C for 30 s. The relative gene expression was calculated using the 2^−ΔΔCT^ method. The primer sequences were detailed in Supplementary Table S2 and were supplied by Prime Biology (Peking, China).

### 2.6 Correlation analysis between prognostic models and clinical characteristics

The correlation between clinicopathological factors and risk models was examined in the TCGA training cohort. Various variables such as age (≤65 or >65 years), sex (female or male), stage (stage I, II, III, or IV), pathologic T (T1, T2, T3, or T4), pathologic N (N0 or N1), pathologic M (M0 or M1), and grading (G2, G3, or G4) were used to categorize the training cohort into different groups. The findings of the correlation analysis were presented through box plots.

### 2.7 Analysis of independent prognostic value of prognostic models

Independent prognostic analyses of clinicopathologic factors were conducted, and risk models were developed using univariate and multivariate Cox analyses. Subsequently, column line plots were created and visualized utilizing the rms R package (Qiu et al., 2022) based on 149 samples from the TCGA training cohort in ccRCC patients. Overall survival (OS) is often the primary observation index of choice in phase III clinical trials and has important clinical significance. Based on this, the index used in survival analysis in this study was OS. Furthermore, the model’s performance was evaluated using calibration curve analysis and ROC curves.

### 2.8 Correlation analysis between risk scores and prognosis-related DICRGs

To better understand the relationship between risk model genes and risk scores, a correlation analysis was conducted using the Pearson, Spearman and Bayes methods, and scatter plots illustrating the correlation as well as histograms depicting the data distribution are created utilizing the R package “ggplot2 (3.2.1)” (Wickham, 2016). Subsequently, the expression levels of prognosis-related DICRGs were compared between the high and low-risk groups by Wilcox. test (P - value <0.05).

### 2.9 Analysis of prognosis-related DICRGs

Using the TCGA and GSE53757 datasets, the expression levels of prognosis-related DICRGs in ccRCC and normal groups were determined using the Wilcox. test. Following the identification of prognostically relevant DICRGs, logistic regression (LR) and support vector machine (SVM) machine learning algorithms were employed to develop diagnostic models in both the TCGA and GSE15641 datasets. These models were subsequently validated in the GSE15641 dataset. Finally, the diagnostic models’ efficacy was assessed using ROC curves.

### 2.10 Identification and functional enrichment analysis of risk-related DEGs

Within the training cohort, comprising 184 high-risk samples and 132 low-risk samples, risk-related DEGs were identified using the limma package (Ritchie et al., 2015) with criteria of P - value <0.05 and |log_2_FC|>1. Volcano plots and heat maps were utilized to visualize the results. Functional enrichment analysis, including GO annotation and KEGG pathway analysis, was conducted using the R package “clusterProfiler” (Wu et al., 2021). The enrichment outcomes were presented visually using the ggplot2 package (Wickham, 2016).

### 2.11 Analysis of immune infiltration and immunophenotyping in high-risk and low-risk populations

The proportion of 22 immune cell types per ccRCC sample in both high-risk and low-risk groups was determined by estimating the relative abundance of RNA transcripts using the cell type identification algorithm Cibersorte (version 1.03) (Chen et al., 2018). Corresponding statistical values were computed, and samples with P - value <0.05 were selected for subsequent analysis. A heatmap illustrating the scores of the 22 immune cell types was generated based on their respective scores. Violin plots were created using the ggplot2 package. The immune phenotype score (IPS) of ccRCC patients was extracted from the TCGA database, and differences in IPS between the high-risk and low-risk groups were assessed. Additionally, correlation analysis was performed using Spearman’s correlation coefficient, and heatmaps were generated using the ggplot2 package (version 3.3.3).

### 2.12 Pharmacovigilance analysis

Risk scores were calculated for the NCI60 cell line in the CellMiner database (60 cell lines), and patients with ccRCC were categorized into high-risk and low-risk groups using the median as the cut-off point. Correlation analysis of federal drug risk scores U.S. Food and Drug Administration (FDA)-approved drugs used in 60 cell lines, IC_50_ was performed using Spearman, |cor|>0.4, P - value <0.05.

## 3 Results

### 3.1 Identification of DICRGs

**FIGURE 1:**
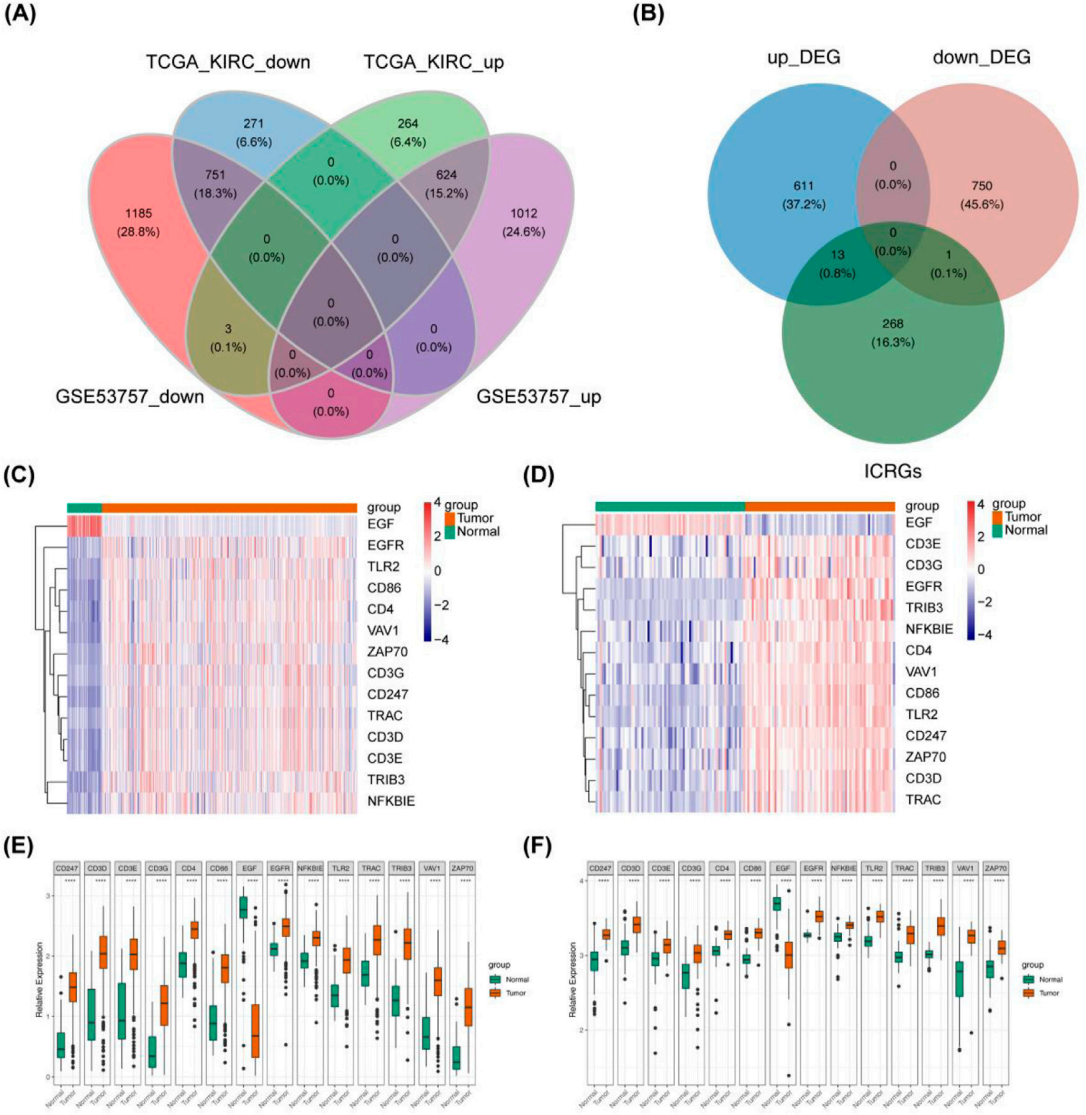
Data Processing and Screening of ICRGs with their Expression Data in ccRCC. **(A)** Venn diagram illustrating 1375 common DEGs, comprising 624 upregulated genes and 751 downregulated genes. **(B)** Venn diagram depicting DEGs and ICRGs, showing 13 upregulated genes and 1 downregulated gene. **(C, D)** Heat map illustrating 14 differentially expressed ICRGs in ccRCC compared with normal tissue in TCGA-KIRC and GSE53757. Red nodes denote significantly upregulated genes with log_2_FC > 1and p < 0.05, while blue nodes represent significantly downregulated genes with log_2_FC < −1 and p < 0.05. **(E, F)** Box plot displaying the expression of 14 differentially expressed ICRGs in ccRCC compared with normal tissue in TCGA-KIRC and GSE53757.

Differential analysis was conducted on extensive RNA sequencing data from TCGA (comprising 530 ccRCC and 72 control samples) and the GSE53757 dataset (with a Tumor: Normal ratio of 72:72). A total of 1,375 DEGs were identified between the ccRCC and Normal groups, comprising 624 upregulated genes and 751 downregulated genes in the ccRCC group (Figure 1A). Differential gene selection criteria included |log_2_fold change|> 1 and P - value <0.05 Subsequently, 14 DICRGs were identified by intersecting DEGs and ICRGs. Among these 14 DICRGs, the expression of 13 genes was upregulated, while 1 gene was downregulated (Figure 1B). Visualization of the expression patterns of the 14 DICRGs in the TCGA and GSE53757 datasets was achieved through heatmaps and box plots (Figures 1C–F).

### 3.2 Construction and evaluation of ICRGs prediction model

**FIGURE 2:**
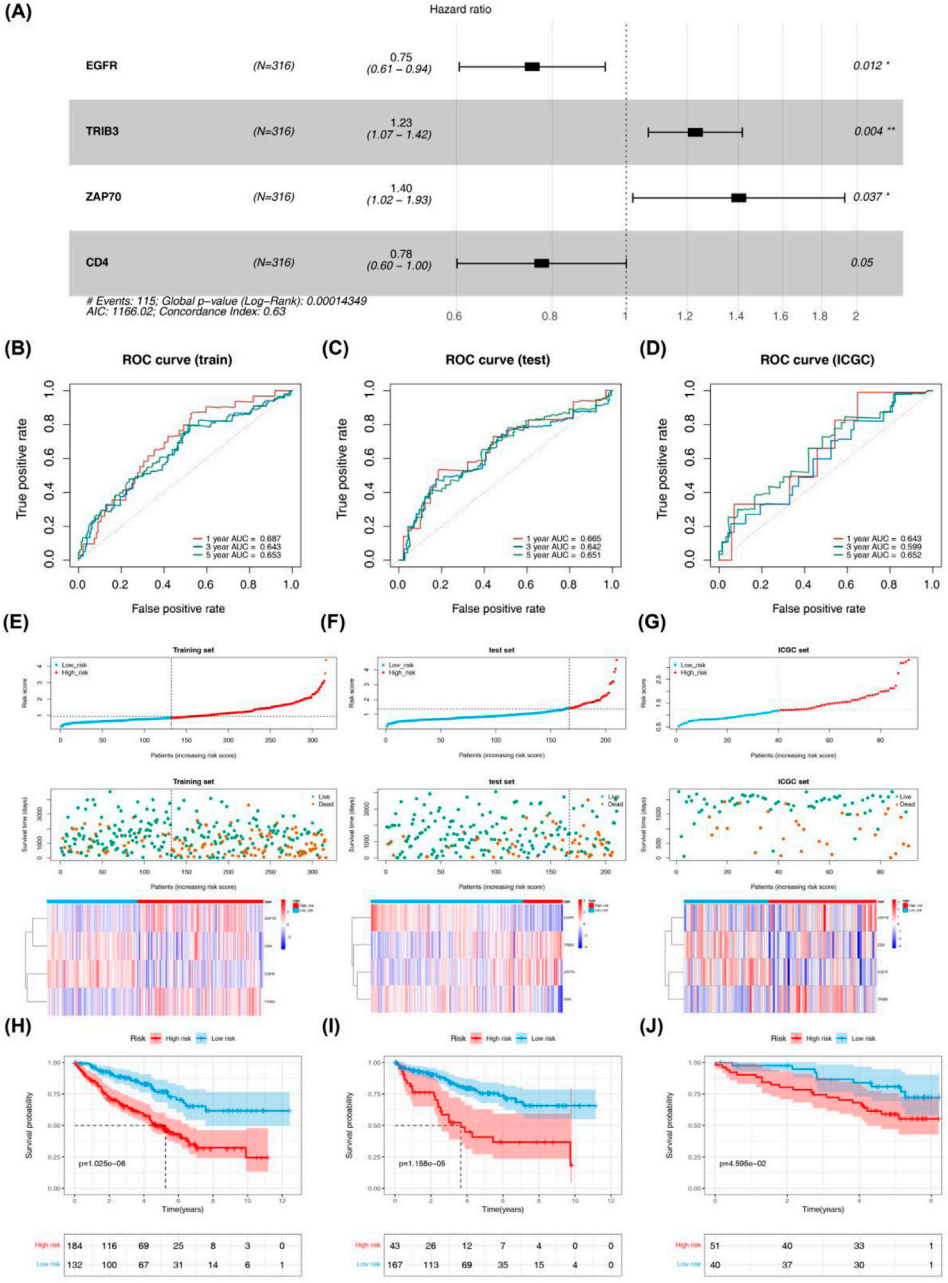
Evaluation and validation of prognostic risk models constructed for four DICRGs in the training set, test set, and ICGC set. **(A)** Forest plot displaying multivariate Cox regression analysis of four prognostically relevant differential ICRGs. The area under the curve (AUC) of time-dependent ROC curves confirms the reliability and accuracy of the risk score in the **(B)** training set, **(C)** testing set, and **(D)** ICGC set. The distribution of the risk score and survival status in the **(E)** training set, **(F)** testing set, and **(G)** ICGC set indicates that higher risk scores correspond to more deceased patients. Heatmaps depict the expression profiles of the four prognostically relevant differential ICRGs between high-risk and low-risk groups in the **(E)** training set, **(F)** testing set, and **(G)** ICGC set. Survival curves illustrate outcomes for high-risk and low-risk groups in the **(H)** training set, **(I)** testing set, and **(J)** ICGC set.

Four DICRGs (EGFR, TRIB3, ZAP70, CD4) prognostically relevant were identified through univariate and multivariate Cox analyses (P - value <0.2) (Table 1; Table 2). Among these, EGFR and CD4 exhibited a protective role in renal cell carcinoma, whereas TRIB3 and ZAP70 were identified as risk factors (P - value <0.05) (Figure 2A). Patients were stratified into high-risk and low-risk groups based on the optimal cutoff values (training cohort = 0.85; test cohort = 1.42; ICGC = 1.18) (Supplementary Tables S3-S5), and risk curves along with heatmaps were generated (Figure 2E). Survival analysis indicated a higher survival rate among the low-risk group in the training cohort (Figure 2H). The ROC curve analysis results for the training cohort demonstrated superior predictive performance of the model, with AUC values exceeding 0.643 at 1, 3, and 5 years (Figure 2B). Subsequently, we evaluated the predictive performance of the model using the test cohort and the external validation cohort. The findings were consistent with those of the training cohort, as evidenced by the risk profile, heatmap, and survival curves displayed for the test cohort (Figures 2F, I). The AUCs for 1-, 3-, and 5-year survival in the test cohort were 0.665, 0.642, and 0.651, respectively (Figure 2C). Similarly, in the external validation cohort, the results were comparable (Figures 2G, J). The AUC values for the 1-, 3-, and 5-year ROC curves in the external validation cohort were all above 0.599 (Figure 2D).

**TABLE 1:** Results from univariate Cox analysis assessing the differential expression of ICRGs.

Gene symbol	HR	HR.95L	HR.95H	Pvalue
*EGFR*	0.725673	0.587673	0.896077	0.002887
*TRIB3*	1.238573	1.073012	1.429679	0.003472
*ZAP70*	1.234896	0.944792	1.614078	0.122517
*CD4*	0.865118	0.703033	1.064573	0.171058

**TABLE 2:** Results from multivariate Cox analysis evaluating the differential expression of ICRGs.

Gene symbol	Coef	HR	HR.95L	HR.95H	Pvalue
*EGFR*	−0.28246	0.753926	0.605501	0.938734	0.011564
*TRIB3*	0.207769	1.230929	1.068747	1.417723	0.003948
*ZAP70*	0.33824	1.402477	1.019827	1.928703	0.037458
*CD4*	−0.25407	0.775637	0.601378	1.00039	0.050352

### 3.3 Expression validation of prognosis-related DICRGs

**FIGURE 3:**
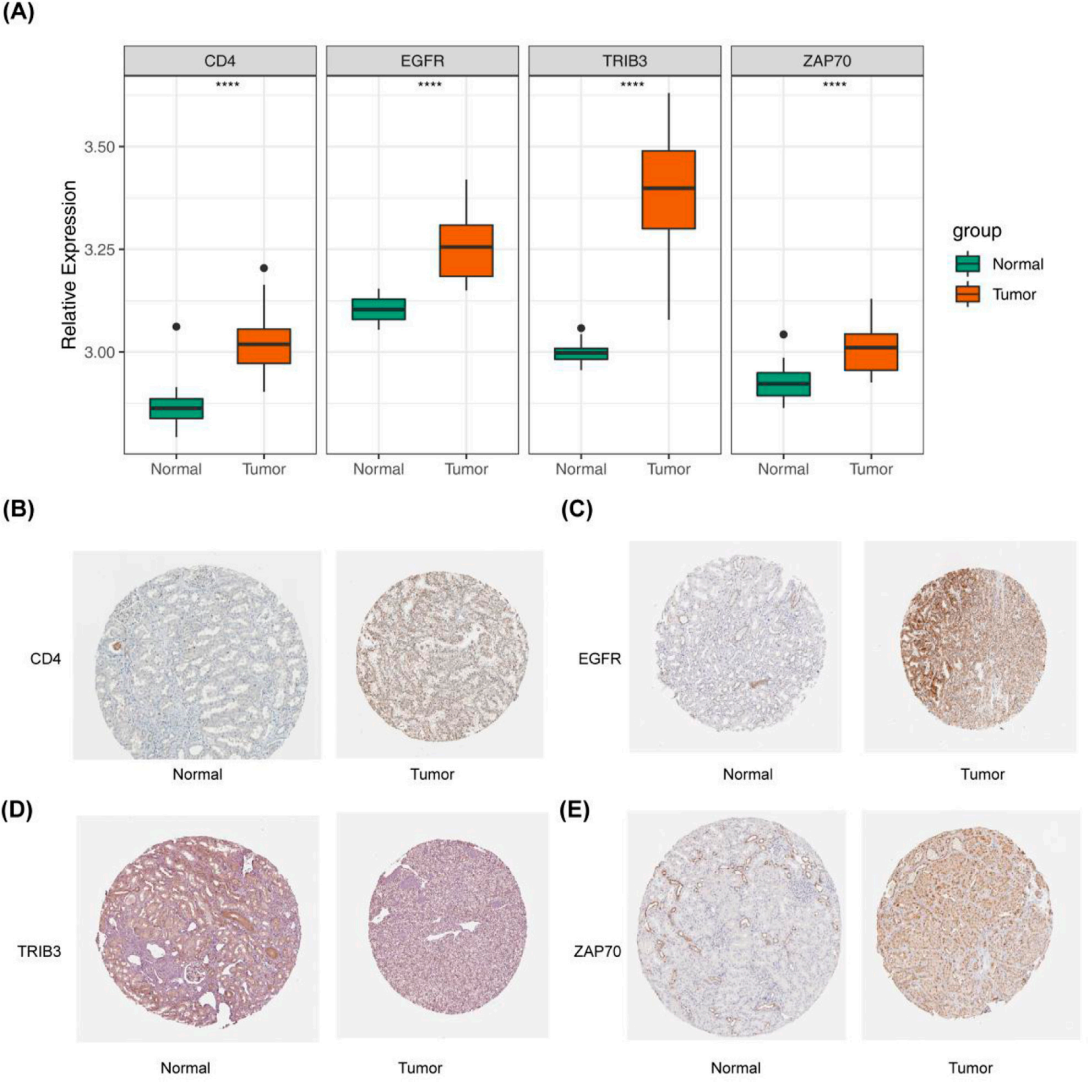
Validation of prognostic models derived from four immune checkpoint-related genes. **(A)** Box plot depicting the expression levels of the four prognostic model genes in the GSE15641 validation set, comparing Normal and Tumor groups. **(B–E)** Immunohistochemical results for the four prognostic model genes in ccRCC tissue. This part of the results is sourced from the HPA database (https://www.proteinatlas.org/). **(B)** Left: *CD4* expression in normal kidney tissue is undetectable in glomerular cells and low in renal tubules. Right: *CD4* shows moderate expression in renal cancer cells, with over seventy-five percent expression. **(C)** Left: *EGFR* expression in normal kidney tissue is moderate in glomerular cells and renal tubules. Right: *EGFR* is highly expressed in renal cancer cells, with over 75% expression. **(D)** Left: *TRIB3* expression in normal kidney tissue is low in glomerular cells and renal tubules, with less than 25% expression. Right: *TRIB3* exhibits moderate expression in renal cancer cells, with 25%–75% expression. **(E)** Left: *ZAP70* expression in normal kidney tissue is low in glomerular cells and renal tubules, with less than 25% expression. Right: *ZAP70* shows high expression in renal cancer cells, with over 75% expression.

In the GSE15641 dataset, the expression levels of the four prognosis-related DICRGs exhibited significant differences between the ccRCC and normal groups, aligning with the expression trends observed in the TCGA dataset (Figure 3A). Within the HPA database, researchers conducted detailed examinations of each protein’s expression across 64 cell lines, 48 human normal tissues, and 20 tumor tissues, utilizing highly specific antibodies alongside immunodetection techniques such as immunoblotting, immunofluorescence, and immunohistochemistry. We conducted a search within the database to retrieve the immunohistochemical results for the four prognostic model genes in ccRCC tissues, presenting the expression patterns in both normal and ccRCC tissues(Figures 3B–E). The immunohistochemical findings revealed elevated expression levels of *EGFR*, *TRIB3*, *ZAP70*, and *CD4* in ccRCC tissues, consistent with the observations from the TCGA database.

### 3.4 Wet bench qRT-PCR for detecting the expression of prognosis-related DICRGs

**FIGURE 4:**
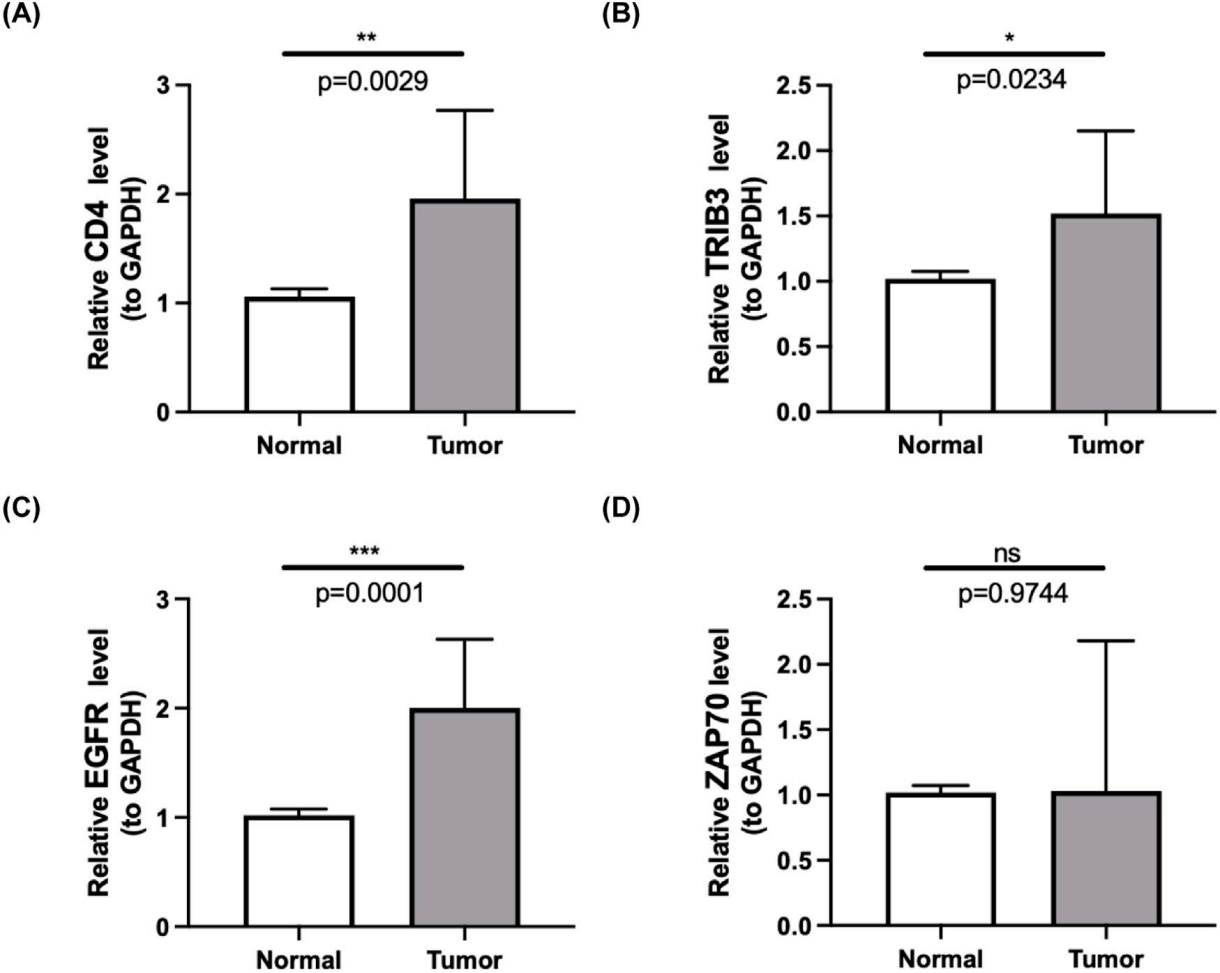
**(A–D)** Quantitative PCR (qPCR) results showcasing the expression levels of four prognostic-related DICRGs in both ccRCC tissues and normal tissues. (n = 10).

To further validate the expression changes of the differentially expressed immune checkpoint-related genes in clinical samples, and to enhance the reliability and accuracy of the research findings, we performed qPCR analysis of the expression differences of EGFR, TRIB3, CD4, and ZAP70 in 10 pairs of ccRCC tumor tissues and their adjacent normal tissues. The results showed that, compared to normal kidney tissues, EGFR, TRIB3, and CD4 were significantly upregulated in ccRCC tumor tissues (p < 0.05), while ZAP70 showed no significant difference (p = 0.9744), suggesting that it may not directly participate in the pathological process of ccRCC. Although these findings provide new insights into the molecular mechanisms of ccRCC, the small sample size (n = 10) and the limitations of qPCR technology require further functional experimental validation. The high expression of genes like EGFR may have a pro-cancer effect and serve as potential biomarkers for ccRCC molecular subtyping and targeted therapy, but larger-scale clinical validation is still needed. (Table 3; Figures 4A–D).

**TABLE 3:** Results from qPCR highlighting the differential expression of ICRGs between tumor tissues and normal tissues.

Gene symbol	Normal	Tumor	P - value
CD4	1.0582 ± 0.0718	1.9585 ± 0.8098	0.0029
EGFR	1.0200 ± 0.0552	2.0016 ± 0.6293	0.0001
TRIB3	1.0202 ± 0.0563	1.5192 ± 0.6329	0.0234
ZAP70	1.0187 ± 0.0530	1.0305 ± 1.1487	0.9744

### 3.5 Subgroup analysis of prognostic model and independent prognostic value

**FIGURE 5:**
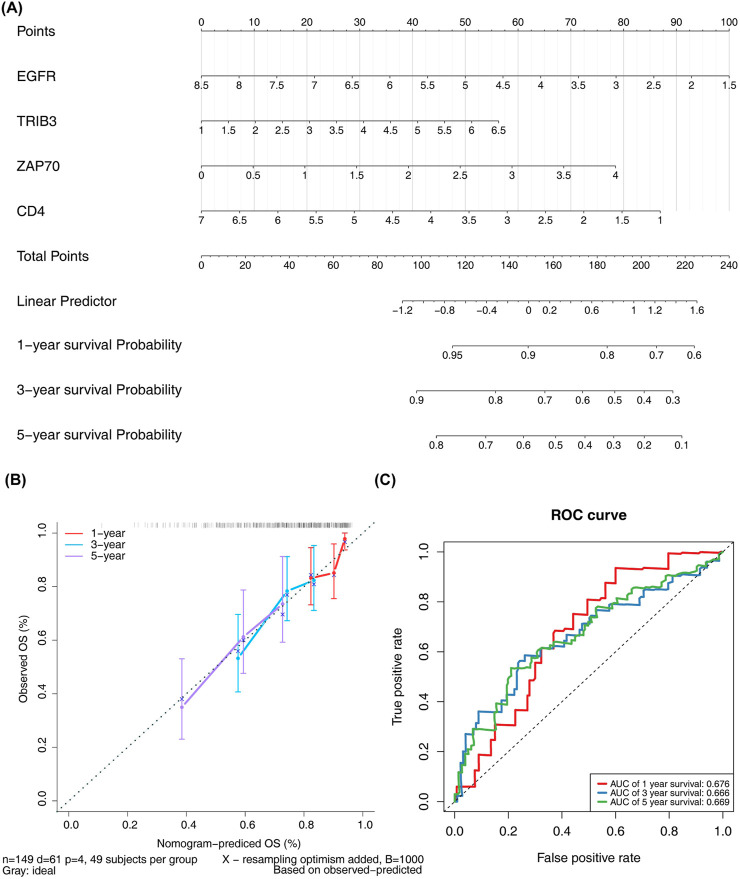
Analysis of independent prognostic value of risk models. **(A)** Nomograms for predicting patient OS based on four prognostic-related DICRGs. **(B)** Calibration curve for the nomograms. A slope closer to 1 indicates more accurate prediction. **(C)** ROC curve for patients with significant clinicopathologic features.

Significant (P - value <0.05) disparities in grading, pathologic T, pathologic M, and stage were detected by comparing the proportions of high-risk and low-risk patients across different subgroups (Supplementary Table S6). The Wilcox test revealed that the risk scores among different subgroups were notably significant (P - value <0.01) for stages G2-G4, T1-T3, and I-IV (Supplementary Figures S1A, B). Moreover, both univariate and multivariate Cox regression analyses demonstrated that the risk score independently influenced the prognosis of ccRCC patients (Supplementary Figure S1C). Column line plots, based on the four prognostically relevant DICRGs, were constructed to forecast patients’ overall survival at 1, 3, and 5 years (Figure 5A). The calibration curves’ slopes at 1, 3, and 5 years approximated 1, indicating a high compatibility between the predictions and actual outcomes (Figure 5B). Additionally, the area under the curves (AUCs) of 0.676, 0.666, and 0.669 were achieved at 1, 3, and 5 years, respectively, suggesting that the risk model exhibited significant prognostic value for ccRCC patients (Figure 5C).

### 3.6 Correlation analysis between risk scores and prognosis-related DICRGs

**FIGURE 6:**
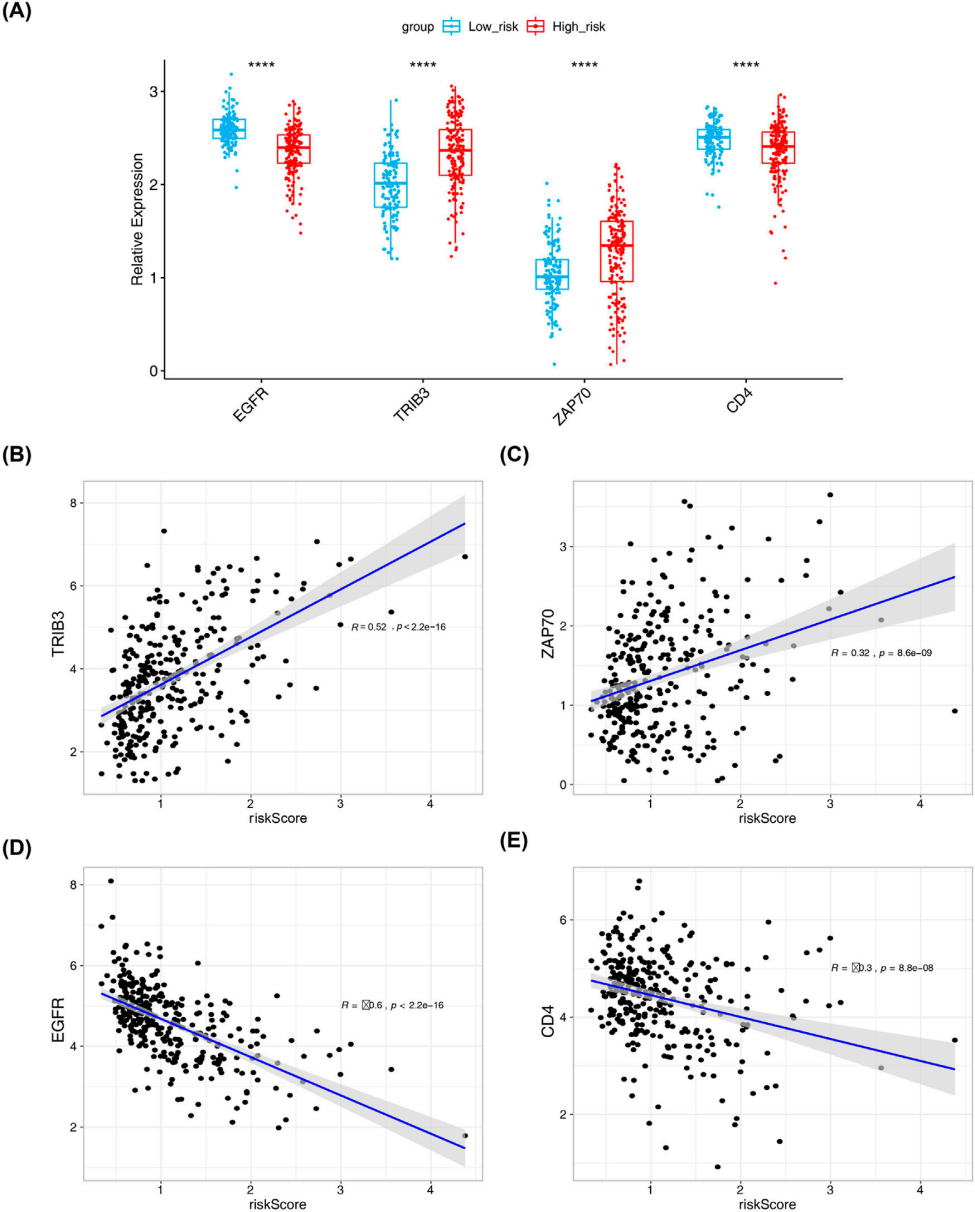
Results from correlation analysis of risk scores with four prognostic model genes. **(A)** Box plot showing expression levels of four risk model genes in high and low-risk groups (Wilcox.test, low risk: n = 131; high risk: n = 184). **(B–E)** Correlation analysis between risk model genes and risk model by pearson method and scatter plot.

A significant disparity in the expression levels of prognosis-related DICRGs was observed between the high-risk and low-risk groups. Specifically, the expression of *TRIB3* and *ZAP70* was markedly higher in the high-risk group compared to the low-risk group, whereas *EGFR* and *CD4* exhibited significantly higher expression levels in the low-risk group than in the high-risk group (Figure 6A). Correlation analysis further revealed a positive correlation between *TRIB3*, *ZAP70*, and risk scores, while *EGFR* and *CD4* showed a negative correlation (Figures 6B–E; Supplementary Figure S2). These findings indicate that TRIB3 and ZAP70 may have a protective role in renal cell carcinoma, whereas *EGFR* and *CD4* could be considered as risk factors.

### 3.7 Construction and evaluation of diagnostic models

**FIGURE 7:**
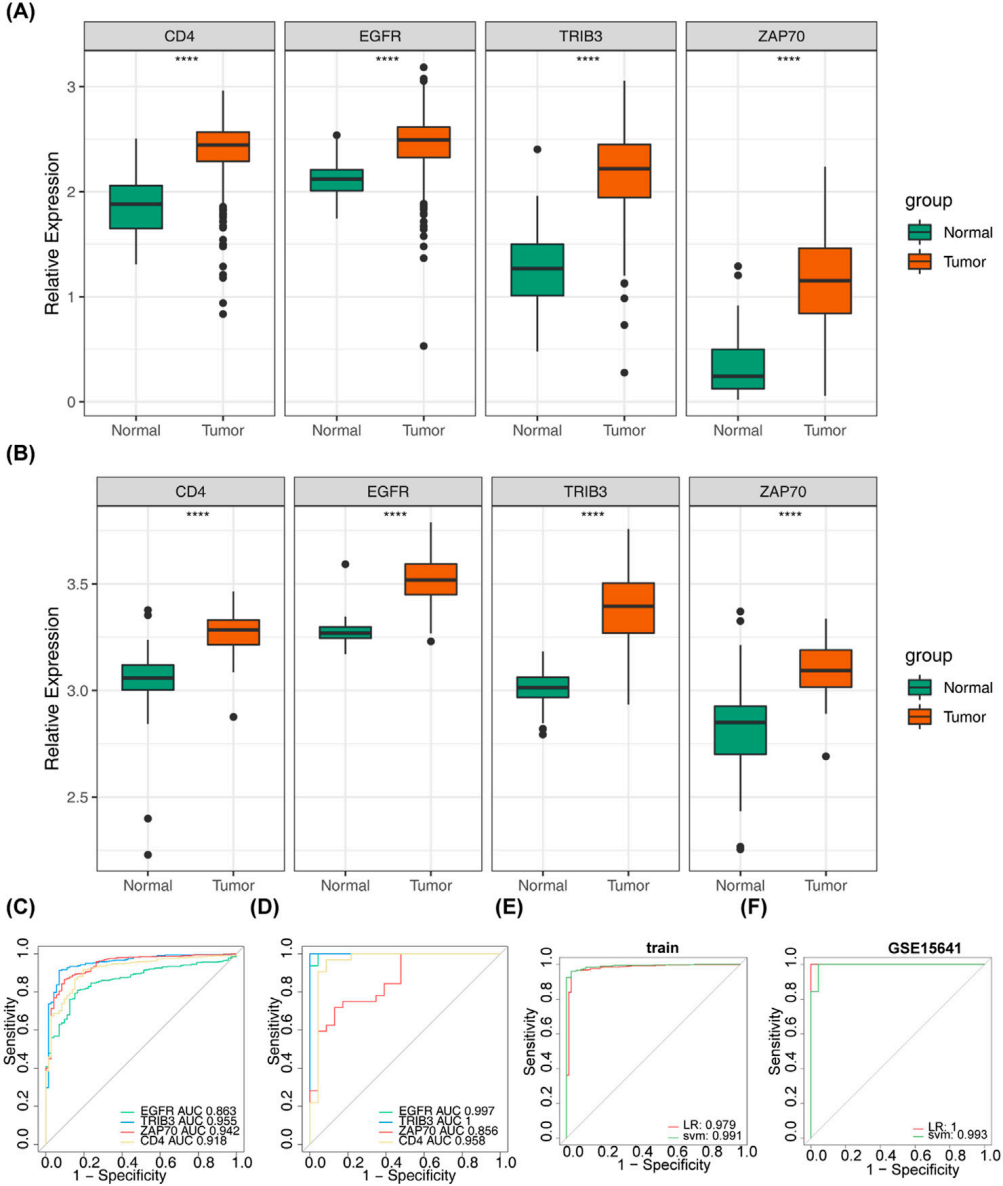
Assessment of the diagnostic value of risk model genes. **(A)** Box-and-line plot showed the expression levels of four prognostic model genes in the normal and tumour groups in the validation set GSE15641. **(B)** Box line plots demonstrated the expression levels of four prognostic model genes in the normal and tumour groups in the dataset TCGA-KIRC. **(C)** Area under the curve (AUC) values of the ROC curves of the four prognostic model genes in the dataset TCGA-KIRC. **(D)** Area under the curve (AUC) values of the ROC curves of the four prognostic model genes in the validation set GSE15641. **(E)** ROC curves were used for assessing and validating the validity of tumour diagnosis (TCGA-KIRC). **(F)** ROC curves were used for assessing and validating the validity of tumour diagnosis (GSE15641).

Following ANOVA analysis, all four prognostically relevant DICRGs within the TCGA and GSE53757 datasets exhibited significant upregulation in the ccRCC group (Figures 7A, B). The ROC curve results indicated that the AUC values of the four prognostically relevant DICRGs within the TCGA and GSE15641 datasets exceeded 0.85, demonstrating their robust diagnostic capability (Figures 7C, D). Subsequently, two machine learning algorithms, LR and SVM, were employed to develop the diagnostic models. The AUC values of these diagnostic models surpassed 0.9, indicating their effectiveness in accurately diagnosing ccRCC (Figure 7E). The validation outcomes within the GSE15641 dataset corroborated the performance of the diagnostic models, with the ROC curves demonstrating AUC values above 0.9 for both diagnostic models (Figure 7F).

### 3.8 Screening and functional enrichment analysis of risk-related genes

**FIGURE 8:**
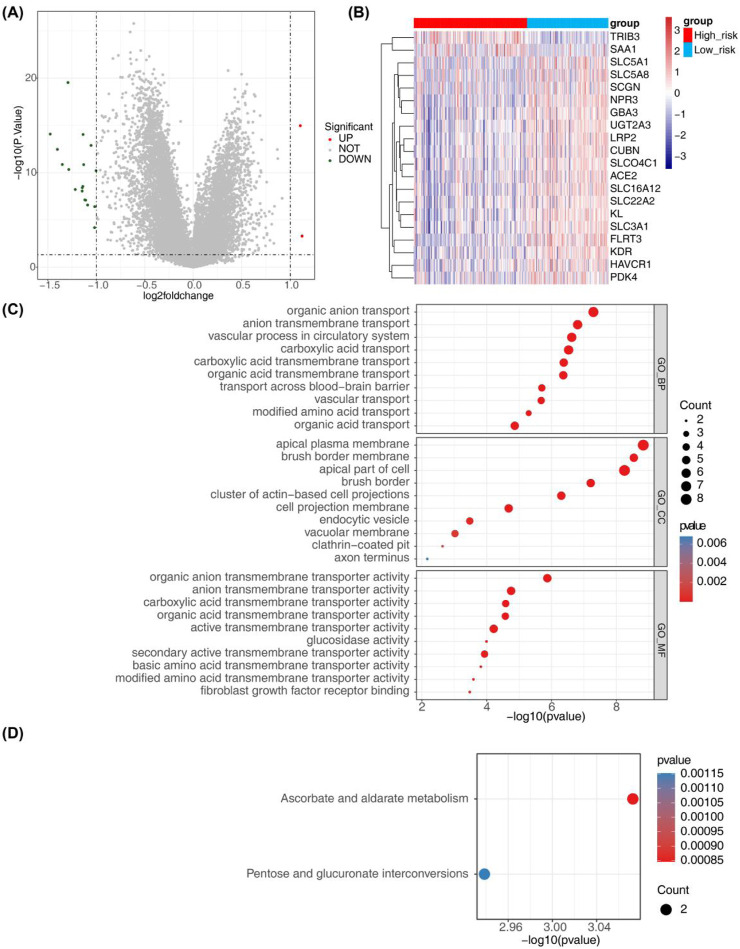
Identification of risk-related DEGs and results from GO and KEGG analyses. **(A)** Volcano plot depicting differential gene expression in the High-risk and Low-risk groups. **(B)** Heatmap illustrating 20 risk-related DEGs. **(C, D)** Results from GO annotation and KEGG functional enrichment analyses of the 20 differential genes in the high-risk and low-risk groups.

A total of 20 risk-associated genes were identified in both the high-risk and low-risk groups, comprising 2 upregulated genes and 18 downregulated genes (Figures 8A, B). The differentially expressed genes (DEGs) were predominantly enriched in terms related to organic anion transport, anion transmembrane transport, and vascular processes within the circulatory system, as illustrated in Figure 8C and detailed in Supplementary Table S7. Moreover, the results of KEGG enrichment analysis revealed significant associations with the metabolism of ascorbic acid and glyoxylate, as well as the inter-conversion of pentose and glucuronide pathways, suggesting potential functional roles of the DEGs (Figure 8D; Supplementary Table S8).

### 3.9 Immune infiltration and immunophenotyping (IPS) public dataset analysis

**FIGURE 9:**
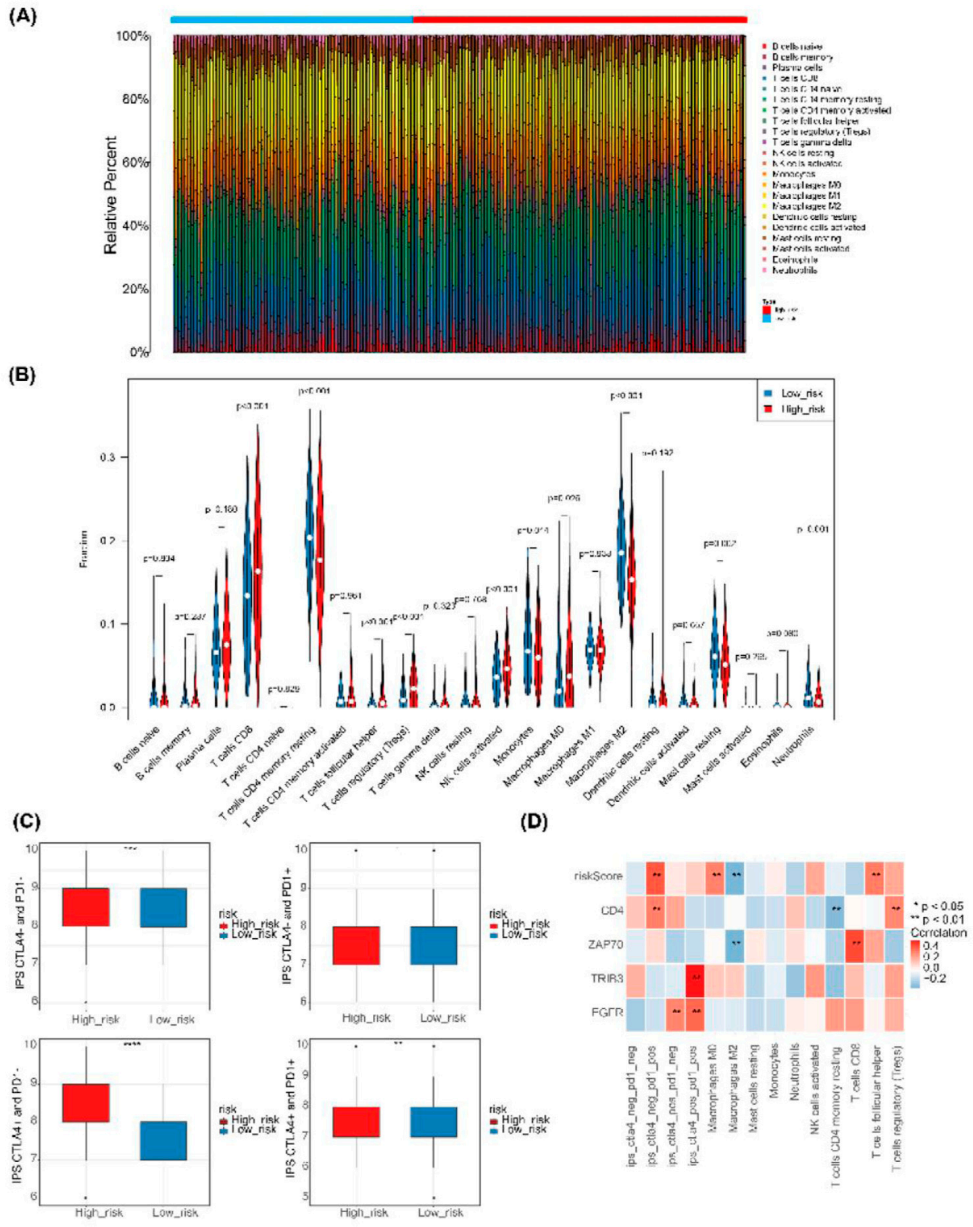
Immune infiltration and immunophenoscore of ccRCC. **(A)** Heatmap depicting the scores of 22 immune cell types in high-risk and low-risk groups. **(B)** Violin plot showing the abundance of 22 immune cell infiltrates in the high-risk and low-risk groups.**(C)** Box plot illustrating IPS expression in high-risk and low-risk groups. **(D)** Heatmap displaying correlations between risk model genes, risk score, differential immune cells, and IPS.

The heatmap in Figure 9A displays the proportion of immune cells in each sample. Ten immune cell types exhibited significant differences (P - value <0.05) between the high-risk and low-risk groups, including CD8 T cells, resting memory CD4 T cells, T follicular helper cells, regulatory T cells (Tregs), NK cells, monocytes, M0 macrophages, M2 macrophages, resting mast cells, and neutrophils (Figure 9B). IPS was notably higher in the high-risk group compared to the low-risk group, as demonstrated in Figure 9C. Correlation analysis revealed that ZAP70 exhibited a positive correlation with CD8 T cells and a negative correlation with M2 macrophages, while CD4 showed a positive correlation with regulatory T cells (Tregs) and a negative correlation with resting memory CD4 T cells. Additionally, risk scores displayed a positive correlation with M0 macrophages and T follicular helper cells, and a negative correlation with M2 macrophages (Figure 9D).

### 3.10 Correlation analysis between drug risk score and IC50

**FIGURE 10:**
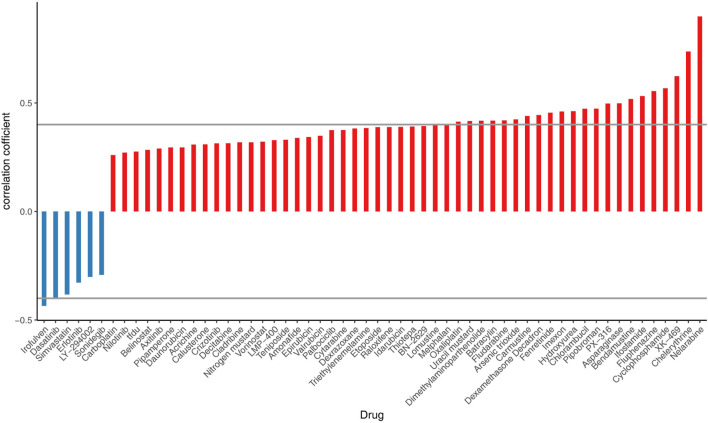
Correlation analysis between chemotherapy efficacy and risk scores in ccRCC patients.

The correlation analysis of cell-related risk scores and IC50 revealed significant associations with 24 drugs (arsenic trioxide, asparaginase, batrachotoxin, bendamustine, carmustine, loratadine, chlorambucil, cyclophosphamide, dasatinib, dexamethasone Decadron, dimethylaminobenzylamine, felitinib, fludarabine, fluphenazine, hydroxyurea, ifosfamide, imefamide, imethadone, inofosfene, nelarabine, oxaliplatin, pipecolonium bromide, PX-316, uracil mustard, and XK-469). These drugs exhibited significant correlations with the prognostic model ((|cor |> 0.4 and P - value <0.05), as illustrated in Figure 10.

## 4 Discussion

Renal cancer, originating from renal tubular epithelial cells (Rini et al., 2009). As cancer cells proliferate, they form tumors that can gradually spread to other parts of the body. According to statistics from 2021, over 76,000 people were diagnosed with kidney cancer, resulting in more than 13,780 deaths attributed to the disease (Siegel et al., 2021). Among renal cell carcinomas, clear cell renal cell carcinoma (ccRCC) is the most common type, accounting for approximately 70%–85% of diagnosed cases (Cohen and McGovern, 2005). Globally, approximately 431,000 new cases of renal cancer and 179,000 new deaths were projected for 2020, with incidence and mortality rates on the rise annually (Sung et al., 2021; Bukavina et al., 2022). Studies have shown that immunotherapy using immune checkpoint modulators is a promising treatment for RCC (Raghubar et al., 2023). Currently, there are no validated prognostic or predictive biomarkers for immunotherapy response in ccRCC patients available for clinical use (Rosellini et al., 2023). Although previous studies have identified several immune checkpoints, including *CTLA4*, *LAG3*, and *PDCD1LG2* (Liao et al., 2021), few have explored the expression patterns of immune checkpoint-related genes (ICRGs) in ccRCC and their prognostic significance.

This study developed a prognostic model that includes four genes: *EGFR, TRIB3, ZAP70*, and *CD4*. Mutations in the *EGFR* gene lead to the abnormal activation of the epidermal growth factor receptor, resulting in continuous cell proliferation and inhibition of apoptosis, which in turn promotes tumorigenesis (Voldborg et al., 1997). Tribble Homolog 3 (*TRIB3*) is a pseudokinase that regulates various intracellular signaling pathways (Wu et al., 2022). Notably, both *EGFR* and *TRIB3* are involved in the MAPK pathway, which plays a pivotal role in cancer development. Their aberrant expression may influence the occurrence and progression of clear cell renal cell carcinoma (ccRCC) through this pathway (Wang et al., 2021; He et al., 2021; Santarpia et al., 2012; Rah et al., 2022; Hong et al., 2019). Univariate Cox analysis in this study indicated that *EGFR* is a low-risk gene for ccRCC (HR = 0.73), suggesting that, during ccRCC progression, the epidermal growth factor receptor may serve functions beyond its tyrosine kinase activity. In contrast, *TRIB3* is a high-risk gene (HR = 1.24). Research has shown that TRIB3 promotes RCC progression by upregulating the lipid droplet-associated protein *PLIN2*. Silencing *TRIB3* expression in RCC cells significantly reduces lipid droplet (LD) accumulation and enhances apoptosis related to endoplasmic reticulum (ER) stress, thereby inhibiting tumor growth and metastasis (Li et al., 2024). In conclusion, the specific mechanisms of EGFR and TRIB3 in ccRCC remain to be fully elucidated.

ZAP70 plays a role in lymphocyte activation and is essential for T-cell receptor (TCR) signaling, while *CD4* is a widely expressed receptor on T-cell surfaces that also participates in the TCR signaling pathway. Both are critical for T-cell development and function (Richardson et al., 2021; Yu et al., 2022; Siu, 2002; Schultz et al., 2022; Gaud et al., 2018). Studies have suggested that *ZAP70* is a potential therapeutic target in the tumor microenvironment (TME) and may influence the prognosis of prostate cancer and bladder cancer (Sun et al., 2021; Kang et al., 2021). CD4^+^ T cells play a crucial role in antitumor immunity by modulating tumor cell lysis and the tumor microenvironment (Melssen and Slingluff, 2017). In patients with renal cell carcinoma (RCC), *CD4* expression is significantly elevated and closely linked to prognosis (Nishida et al., 2020). qRT-PCR results revealed that *CD4* expression was markedly upregulated in the disease group, consistent with previous studies. In contrast, *ZAP70* expression did not show a significant statistical difference, which may be attributed to the limited sample size and the fact that the samples were exclusively from Asian populations. However, data on protein expression levels from the HPA database indicate that *ZAP70* expression is higher in cancer tissues than in normal kidney tissues. Therefore, further research with a larger sample size is necessary to explore the potential relationship between *ZAP70* and ccRCC in greater depth.

Diagnostic models are now widely utilized in cancer research (Tang et al., 2021; Guo et al., 2023). The model demonstrates remarkable potential for the early diagnosis and prognostic assessment of cellular carcinoma. Its clinical applications extend beyond diagnosis, providing personalized treatment guidance for patients. When combined with physicians’ professional judgment, the model is expected to become a vital tool in the diagnostic and treatment processes for renal cancer, enhancing treatment outcomes and improving patients’ quality of life (Guo et al., 2023). In this study, the area under the curve (AUC) for the four prognostic model genes exceeded 0.8, and the overall model’s AUC was greater than 0.65, confirming that the model we developed demonstrates strong diagnostic accuracy and holds significant clinical implications. Wang et al. (Wang et al., 2023) constructed a prognostic model for lung adenocarcinoma using LASSO, which yielded an AUC around 0.6. Similarly, Zhang et al. (Zhang et al., 2021) developed a prognostic model for colorectal cancer using both univariate and multivariate Cox regression, with an AUC also around 0.6. In contrast, our study incorporated stepwise regression analysis in multivariate Cox regression and adjusted the multivariate model, thereby enhancing the model’s robustness and improving its predictive performance.

ccRCC patients were subsequently stratified into two subgroups based on four prognostically relevant Immune Checkpoint-Related Genes (ICRGs). Our comparative analysis between these subgroups identified 20 differentially expressed genes, such as *SAA1*. These genes were primarily associated with organic anion transport, ion transmembrane transport, vascular processes in the circulatory system, ascorbic acid and glucuronic acid metabolism, and interconversion of pentose and glucuronic acid. Xu et al. demonstrated that *SAA1* may serve as a novel marker for predicting the prognosis of ccRCC patients and may also be expressed in the tumor microenvironment (TME) through mast cell resting and PDL1 expression. *SAA1* holds potential as both a therapeutic target and an indicator for immune and targeted therapies in ccRCC treatment (Xu et al., 2023). Wei et al. reported that SLC-related genes (e.g., genes such as *SLC5A1*, *SLC3A1*, etc.) are correlated with predicting prognosis in ccRCC, indicating their role in the immune environment, and suggesting SLC-related genes as promising therapeutic targets (Bao et al., 2023; Wang and Zou, 2020). Lai et al. demonstrated that ccRCC patients with high SCGN expression may have a better prognosis. Their results revealed that the percentage of SCGN high-expression in primary foci of patients with metastatic renal cell carcinoma was significantly lower than that of patients with limited renal cell carcinoma (Lai et al., 2023). In a study by Gremel et al. (Gremel et al., 2017) ccRCC patients with CUBN-positive tumors had a significantly better prognosis than patients with CUBN-negative tumors, irrespective of T-stage, Fuhrman grade, and lymph node status. Some investigators have proposed that KL serves as a valuable immune-related prognostic factor for ccRCC, with its downregulation in ccRCC tissues indicating disease progression and shorter overall survival (Pan KH. et al., 2023).

There is evidence that immune cells within the tumor microenvironment play a crucial role in renal carcinogenesis and in the resistance to immune checkpoint inhibitors (Lai et al., 2021; Pan Y. et al., 2023). In this study, we investigated the infiltration of immune cells in high- and low-risk groups. The results identified ten types of immune cells that exhibited significant differences between these groups. Furthermore, correlation analysis revealed a significant positive correlation between CD8 and *ZAP70*, as well as a significant negative correlation between risk scores and M2 macrophages. Additionally, it has been suggested that CD8 T cells enhance cancer cell membrane permeability and promote cell death (Raskov et al., 2021), and their high infiltration has been associated with improved tumor therapy outcomes (Nalio Ramos et al., 2022; Jiang et al., 2020). However, in renal carcinoma, high levels of CD8 T cell infiltration are correlated with poor prognosis (Qi et al., 2020), which aligns with the findings of this study. This suggests that CD8 T cells may have a specific role in the progression of renal cell carcinoma. James et al. (James and Vale, 2012) elucidated that *ZAP70* is critical for T-cell receptor signaling, speculating that it may synergistically influence the progression of renal cell carcinoma. Previous studies have demonstrated that high permeability memory quiescent CD4 T cells and M2 macrophages are associated with better outcomes (Zhang et al., 2019). In contrast, M0 macrophages have been linked to poor prognosis (Pan et al., 2020; Tao et al., 2021), and the infiltration patterns of these immune cells in renal cell carcinoma align with the findings presented here. M2 macrophages were highly infiltrative in the low-risk group and showed a significant negative correlation with the risk score, which contrasts with the MOST results. The polarized state of macrophages may be associated with regulatory T cells (Tregs), potentially leading to tumor immune escape by hindering the function of CD4 T helper cells and the production of tumor-specific CD8 cytotoxic T lymphocytes (CTLs) (Li et al., 2020). Tregs can also diminish the efficacy of immune checkpoint inhibitors (ICIs). Targeting CD8^+^ T cells shows promise in enhancing anti-tumor immune responses, while modulating the function of regulatory T cells (Tregs) can mitigate their suppressive effects on the immune response. This dual approach may effectively reverse tumor immune escape and enhance the clinical efficacy of immunotherapy. The impact of the tumor immune microenvironment on renal cell carcinoma arises from the interactions of multiple immune cells and necessitates a comprehensive analysis.

Finally, we analysed the correlations between risk scores and center of inhibition values were analyzed for FDA-approved drugs across 60 cell lines. The results indicated that 24 drugs were significantly associated with the risk model (|cor| > 0.4 and p < 0.05), suggesting that high-risk individuals may exhibit increased sensitivity to these drugs. However, it is important to note that there are currently no established recommendations for chemotherapy in advanced ccRCC. Nonetheless, we may explore this area in the future.

In this study, we constructed a prognostic model for ccRCC patients based on immune-related genes (ICRGs: EGFR, TRIB3, ZAP70, CD4) using transcriptomic data from the TCGA and other databases. This model has the potential to serve as a biomarker for exploring the molecular mechanisms associated with ccRCC prognosis. Additionally, it may facilitate early lesion identification, subtype classification, and adjunctive non-invasive screening, offering insights for future therapeutic strategies for ccRCC. However, this study has several limitations. First, since the model validation in this study primarily relies on public databases and has not been tested on independent, private datasets, the generalizability of the findings may be somewhat limited. In future research, we aim to increase the sample size and conduct more comprehensive validation to improve the statistical power and broader applicability of the results. Second, although this study has identified several genes, their biological functions in renal cancer cell lines have yet to be fully explored. We plan to expand research in this area moving forward. In addition, although we have conducted qRT-PCR experiments for experimental validation, the limited sample size has been a constraint. Furthermore, some studies have indicated that GAPDH expression may be elevated in cancer samples (Mori et al., 2008). Therefore, in future studies, we plan to not only increase the sample size but also explore more stable reference genes, such as β-actin or 18S rRNA, to evaluate the expression differences of prognosis-related DICRGs between normal and disease samples. Our future goal is to strengthen the model’s predictive and interpretative capacity by incorporating additional clinical parameters, conducting meta-analyses to integrate multiple datasets, and incorporating clinical variables. We also intend to carry out histological analysis using our own data, alongside immunohistochemistry, cell-based experiments, gene editing, and other assays, to further validate and explore the functional roles of prognostic genes, thereby deepening our understanding of their underlying mechanisms. In summary, we have developed a promising prognostic model for ccRCC patients based on ICRGs using transcriptomic data from the TCGA database. This preliminary study offers new insights into the treatment of ccRCC and the investigation of molecular mechanisms associated with its prognosis. However, the findings and conclusions of this study warrant further exploration of potential mechanisms and molecular validation.

## Data Availability

The datasets presented in this study can be found in online repositories. The names of the repository/repositories and accession number(s) can be found in the article/Supplementary Material.
