# Supplementary material for: Development and validation of prognostic and diagnostic models utilizing immune checkpoint-related genes in public datasets for clear cell renal cell carcinoma
Source: Front Genet. 2025 Mar 4;16:1521663. doi: 10.3389/fgene.2025.1521663 (PMC11913831; doi:10.3389/fgene.2025.1521663)
Supplement: Supplementary file 2 [file Table1.docx]

# Supplementary Table 1 RNA Concentration Detection Results

| Serial Number | Sample Name | volume（µl） | concentration(ng/µl) | A260/A280 | A260/A230 |
| --- | --- | --- | --- | --- | --- |
| 1 | ZB | 30 | 1918.9 | 1.852 | 1.504 |
| 2 | K6 ZB P | 30 | 2160 | 1.922 | 2.216 |
| 3 | K9 ZB P | 30 | 297.36 | 1.73 | 1.235 |
| 4 | K4 ZB 2 | 30 | 2725.6 | 1.922 | 2.222 |
| 5 | K5 ZB 2 | 30 | 418.88 | 1.961 | 1.519 |
| 6 | K2 ZB 2 | 30 | 1704.10 | 1.933 | 2.038 |
| 7 | P6 ZB | 30 | 404.08 | 1.595 | 1.300 |
| 8 | K3 ZB 2 | 30 | 355.28 | 1.812 | 1.559 |
| 9 | ZB K12 P | 30 | 1729.5 | 1.981 | 2.078 |
| 10 | ZB K13 P | 30 | 431.44 | 1.997 | 1.454 |
| 11 | K6 Z1 | 30 | 330.64 | 1.889 | 0.989 |
| 12 | K6 ZB T | 30 | 966.2 | 1.545 | 1.062 |
| 13 | K9 ZB T | 30 | 477.40 | 1.864 | 1.608 |
| 14 | K4 ZB 1 | 30 | 434.8 | 1.757 | 1.492 |
| 15 | K5 ZB 1 | 30 | 1481.4 | 1.509 | 1.585 |
| 16 | K2 ZB 1 | 30 | 1985.8 | 1.828 | 2.047 |
| 17 | K6 ZB | 30 | 988.4 | 1.680 | 0.611 |
| 18 | K3 ZB 1 | 30 | 1265.6 | 1.565 | 1.287 |
| 19 | ZB K12 T | 30 | 744.28 | 1.538 | 1.858 |
| 20 | ZB K13 T | 30 | 497.84 | 1.737 | 1.367 |
