# Supplementary material for: Development and validation of prognostic and diagnostic models utilizing immune checkpoint-related genes in public datasets for clear cell renal cell carcinoma
Source: Front Genet. 2025 Mar 4;16:1521663. doi: 10.3389/fgene.2025.1521663 (PMC11913831; doi:10.3389/fgene.2025.1521663)
Supplement: Supplementary file 4 [file Table2.docx]

**Supplementary Table 2** Primer sequences associated with the qRT-PCR experiment

| **primer** | **sequence** | |
| --- | --- | --- |
| CD4 F | CGGATTGACTGCCAACTCTGAC |  |
| CD4 R | CCACCTGTTCCCCCTCTTTCTT |  |
| EGFR F | GGTGAGCCAAGGGAGTTTGT |  |
| EGFR R | CGTCCTGTGCAGGTGATGTT |  |
| TRIB3 F | GCGGTTGGAGTTGGATGACA |  |
| TRIB3 R | GCACGATCTGGAGCAGTAGG |  |
| ZAP70 F | TGTCTGGAGCTATGGGGTCA |  |
| ZAP70 R | AGTGCGTACAGTTCGGGTGG |  |
| internal reference-GAPDH F | CGAAGGTGGAGTCAACGGATTT |  |
| internal reference-GAPDH R | ATGGGTGGAATCATATTGGAAC |  |
